# Who participates in value-based care models? Physician characteristics and implications for value-based care

**DOI:** 10.1093/haschl/qxae087

**Published:** 2024-07-16

**Authors:** Debra R Winberg, Matthew C Baker, Xiaochu Hu, Keith A Horvath

**Affiliations:** Health Care Affairs, Association of American Medical Colleges, Washington, DC 20001, United States; Department of Health Policy and Management, Tulane University School of Public Health and Tropical Medicine, New Orleans, LA 70112, United States; Health Care Affairs, Association of American Medical Colleges, Washington, DC 20001, United States; Health Care Affairs, Association of American Medical Colleges, Washington, DC 20001, United States; Health Care Affairs, Association of American Medical Colleges, Washington, DC 20001, United States

**Keywords:** value-based care, alternative payment models, payment models, accountable care, bundled payments, capitation

## Abstract

Value-based care (VBC) payment models are becoming increasingly prevalent as alternatives to the traditional fee-for-service paradigm. This research quantifies the relationship between physician characteristics and participation in VBC payment models using the Association of American Medical Colleges’ 2022 National Sample Survey of Physicians. We specified logistic regressions using physician-level variables to assess associations with current and new participation in Accountable Care Organizations, Primary Care First model, capitation, and bundled payments. Our results indicate that most respondents engaged in at least 1 VBC. Participation varied based on several characteristics, and physician specialty was highly predictive of overall participation. Compared with primary care physicians (PCPs), hospital-based physicians (odds ratio [OR] = 0.6, *P* < .001), medical specialists (OR = 0.5, *P* < .001), psychiatrists (OR = 0.4, *P* < .001), and surgeons (OR = 0.5, *P* < .001) were less likely to participate in VBC models. Medical specialists and surgeons were less likely to participate in commercial capitation than PCPs, while medical specialists and obstetricians/gynecologists were more likely to participate in certain bundles than PCPs. We suggest several policies to close the cross-specialty participation gap by including specialists and appealing to providers and patients.

## Introduction

In the United States, value-based care (VBC) payment models are gaining popularity over traditional fee-for-service (FFS) models as a way to improve population health and contain rising health care costs. To help enable this transition, the Centers for Medicare and Medicaid Services (CMS) and other payers have adopted several policies and payment strategies. Significant model types include bundled payments, Accountable Care Organizations (ACOs), the Medicare Shared Savings Program (MSSP), and others that have evolved since the Affordable Care Act (ACA) launched the Centers for Medicare and Medicaid Services Innovation Center (CMMI) in 2010.^1,2^ By focusing on overall cost and quality outcomes rather than individual services, VBC models intend to encourage a more efficient, multifaceted approach to health care delivery, ultimately lowering health care costs.^3^

The structure of these progressive payment models varies from population-based models, such as ACOs and capitated payments, to episodic payments (eg, bundled payments). The associated financial and other incentives aim to increase the quality of care while simultaneously lowering costs by encouraging coordinated care, decreasing service duplication, and offering physicians greater flexibility to provide patients with the right care at the right time.^4^ Evidence has begun to emerge showing the wide-reaching VBC effect: the ACO-based Medicare Shared Savings Program alone achieved $2.3 billion in savings in 2020,^5^ while primary care physicians (PCPs) compensated through capitation models reported higher rates of patients completing crucial screenings.^6^ However, these successes have come at a tradeoff, and researchers have identified adverse effects, including increasing burnout.^7^ A recent report illustrating that CMMI increased direct spending by over $5 billion in its first 10 years, rather than decreasing costs, underscores these potential negative impacts.^8^ Policymakers and payers must focus on mitigating these adverse effects and learn to overcome barriers to participation to ensure VBC does not stall.^4,9,10^ As addressed in this research, understanding the current state of payment models can improve VBC beyond the current phase.

Multiple factors limit participation below CMS's 2030 universal participation goal,^11^ including model design, health system–level influences, and employer-level factors. Political and system-level factors drive participation by operating as a catalyst, incentivizing the adoption of innovative payment models while introducing mandatory structures to shape the landscape, as the ACA did. At the more granular level, individual physician decisions (eg, specialty choice and practice location) determine the physician's exposure to these influences to the extent that a model targets specific subsets of physicians. Increasing the understanding of how physician characteristics influence exposure to participation opportunity will be essential as policies promoting VBC develop, and new models are built.

Current estimates of VBC participation rates vary. In 2018, only 45% of US physicians were estimated to utilize at least 1 Medicare payment model, while between 2017 and 2022, 66% to 91% of physician practices participated in at least 1 VBC model.^12-14^ Differences in reported participation arise due to methodological challenges in the current literature, including insufficient sample sizes that may bias results. Additionally, the definition of VBC models differs between studies, and some studies include models such as pay for performance, which are intermediate steps to VBC.^15^ A subsection of research analyzes the system- and physician-level characteristics which may explain differences in participation. For example, PCPs may report participation at higher rates because they are more likely than specialists to understand the purpose of VBC models such as ACOs, know if they are part of an ACO, and have more positive views of VBC models, including ACOs, than specialists.^16,17^ As recent research highlights that ACOs with a higher proportion of PCPs have greater savings,^18^ it is important to fully grasp these differences in participation.

This research aims to increase the understanding of VBC participation by quantifying the relationship between physician characteristics and participation in a wide range of VBC models using national physician data. Filling this research gap provides support for policymakers to design tailored, comprehensive VBC payment models and increase the quality of patient care.

## Data and methods

### Data

This study analyzes data from the Association of American Medical Colleges’ (AAMC's) 2022 National Sample Survey of Physicians (NSSP).^19^ The NSSP, a recurring nationally representative survey of US physicians, encompasses demographic details, practice characteristics, employment characteristics, and other data points such as academic affiliations, work time, and telehealth. The 2022 NSSP was collected between May and November 2022, consisting of 5917 active physicians and included questions like telehealth utilization and payer mix. A detailed NSSP sampling methods documentation was published online.^19^ The American Institutes for Research Institutional Review Board reviewed and approved the survey.

### Dependent variables (VBC payment model participation)

One of the 2022 NSSP questions asked respondents, “In which of the following alternative payment models do you participate? And in which did you participate 3 years ago?” Respondents indicated which payment models they participated in: Medicare ACO, commercial ACO, Medicaid ACO, Primary Care First (PCF) models,^20^ commercial capitation, commercial bundles, or Medicare bundles. Additionally, respondents could select “none” or “other” and describe which model(s) they used. Respondents qualified as VBC participants when they indicated using at least 1 of the 7 payment models. These models were included in the survey because they are available on a national level and able to be scaled. If respondents chose “not applicable” or did not answer the current column but selected 1 of 7 models for participation 3 years ago (in 2019), they were considered non-VBC participants. We uniformly applied the criteria for current and past participation in each model. For each model, we classified respondents as a “consistent participant” (participated in 2019 and 2022), “current participant” (participated in 2022 but not 2019), “former participant” (participated in 2019 and not 2022), or “consistent non-participant” (did not participate in 2019 or 2022). Although pay-for-performance models may serve as intermediary steps to VBC models, their incentive structure may not always be as broad as other VBC models and were therefore not considered in the survey.

Dependent variables included the payment model prevalence participation, the likelihood of participating in 2022, the likelihood of newly participating in 2022 if they did not participate in 2019, and the intensity of participation. Intensity of participation is measured as a count of the number of VBC model types among the following categories, which aggregate different payers from our survey data: ACOs, bundled payments, capitation, and PCF models regardless of payor. We repeated the analysis for each model to assess if specific models were driving results. We computed 1 additional version of participation by model type, rather than by specific model, grouping similar models across payers (such as commercial and Medicare ACOs) to allow us to measure whether participation in a specific model from 1 payer was associated with participation in the same model from another payer (Appendix 2). (To access the Appendix, click on the Details tab of the article online.)

### Independent variables (physician characteristics)

We assessed several physician-level, explanatory variables for each outcome described above, with a respondent's specialty as the primary variable of interest. Respondents reported practicing in 1 of over 200 specialties using a drop-down list. We then cross-walked these specialties to 65 specialty categories from the CMS and then grouped the 65 specialties into 1 of 7 specialties: primary care, hospital-based, medical specialty, gynecology/obstetrics, psychiatry, surgery, or other.^21^ We chose these groupings, rather than more granular specialty categories, to ensure an appropriate sample size for analysis. Primary care referred to general practice, family practice, internal medicine, pediatric, geriatric, hospice, and preventative medicine. We separated hospital-based physicians from other specialties because of their distinct patient population and procedures, which affects their propensity to participate in payment arrangements.

We included race and ethnicity, gender, age, affiliation with a teaching hospital or medical school, census region, time spent in rural areas, payer mix, place of practice, employer, and the time spent in patient care. Respondents could check boxes for each race they identified with, including the following: Hispanic/Latino/or of Spanish origin, American Indian or Alaskan Native, Asian, Black or African American, Native Hawaiian or Other Pacific Islander, White, or Other. We categorized these into Hispanic, Latino, or of Spanish origin, Asian, Black or African American, White, Other - Mixed (if checked multiple boxes), Other (if answered Other, American Indian/Alaskan Native or Native Hawaiian/Pacific Islander, or Unknown if they did not answer. These categories were created due to small sample sizes in several response categories. Respondents self-reported time spent in rural areas as a percentage, which we then categorized into “primarily urban or suburban” (0%-20% of time in rural areas), “mixed urban/rural time” (21%-80% of time in rural areas), or “rural serving” (81%-100% of time in rural areas). The cutoff was based on the distribution of the variable, which is U-shaped: nearly 80% of the respondents reported 0% in rural and 8% reported 100% time in rural. In the rather flat distribution in the middle, there are 2 models at 10% and 20%. We decided to use 20% as the rural–non-rural cutoff to include more respondents into the “rural” category and create a more even distribution of the rural vs non-rural categories. We calculated the payer-mix variable from the self-reported commercial insurance, Medicaid, Medicare, dual insurance, or no insurance distributions. We then grouped these into categories for “safety net” (Medicaid, dual-eligible, and uninsured), “Medicare,” “mixed,” and “commercial.” A respondent belonged to a discrete payer category if they saw at least 50% of patients from 1 category. Otherwise, we categorized them as mixed payers.

Employee status indicates whether respondents are employees, owners, independent contractors, or others. Respondents answered several yes/no questions about the types of health care entities where they worked. We defined practice location based on a respondent's response to where they worked and categorized answers as “outpatient,” “emergency/urgent care,” or “long-term care.” We classified respondents as either spending most of their time in 1 location or most of their time in “mixed” locations. Second, respondents selected their employment arrangement, which we then categorized into 1 or more of the following work settings: health system, private practice, hospital, group, other, or in multiple categories if they chose “yes” for multiple settings. Employer categories are not mutually exclusive.

Participants reported whether they currently use each of the following 6 modalities of telehealth technology in providing patient care: (1) electronic patient communications, (2) live video visits with patients, (3) billable telephone visits with patients, (4) remote patient monitoring, (5) electronic asynchronous consultations with other physicians, and (6) live video consultations among physicians. We classified the first 4 modalities as provider-to-patient and the last 2 as provider-to-provider, then categorized physicians into 4 groups based on their current telehealth use as follows: (1) non-user, (2) provider-to-patient only, (3) provider-to-provider, and (4) dual-users. Additional details on the specific measurement and instrument for physician characteristics are provided in Appendix 1.

Our analytical sample contained 5268 respondents and excludes those who worked part-time, defined as fewer than 30 hours per week, or those who did not answer the question on payment models for 2019 and 2022.

### Statistical analysis

We conducted logistic regressions to assess physician characteristics associated with (1) current participation, (2) new participation since 2019, and (3) model-specific participation. We also conducted a linear regression model to assess the number of models in which a physician participated. We adjusted each model for the control variables described above. We applied weights to correspond to the distribution of US physicians identified in the American Medical Association (AMA) Physician Characteristics database.^19^ Stata 14.0 (2015; Stata Statistical Software: release 14; StataCorp LLC, College Station, TX) was used for statistical analysis.

### Limitations

This study has several limitations. First, respondents self-reported their answers through a survey, and answers were unverified. Self-reported data may also suffer from recall bias, as responses were collected in 2022 about participation 3 years prior. Value-based care participation self-reports can be unreliable because physicians may not know their organization participates in certain models, which may introduce bias. To counter the most unreliable responses, our analysis omitted the “other payment model” category because respondents responded with models that are not always used to support VBC. Moreover, we included only frequently utilized payment models, and participants may participate in other models not measured in this study. Similarly, the initial “other” category was needed as the NSSP did not include an exhaustive list of VBC models and some may be missing from the study, thus underestimating our VBC participation estimates. Additionally, participation may be sensitive to the grouping and granularity of each model type; some models, such as Medicare bundled payments, could have been split into mandatory and voluntary. Last, although we include data from 2019, this research is cross-sectional and we cannot ascertain causal relationships between our dependent variables and VBC participation.

## Results

**Figure 1.**
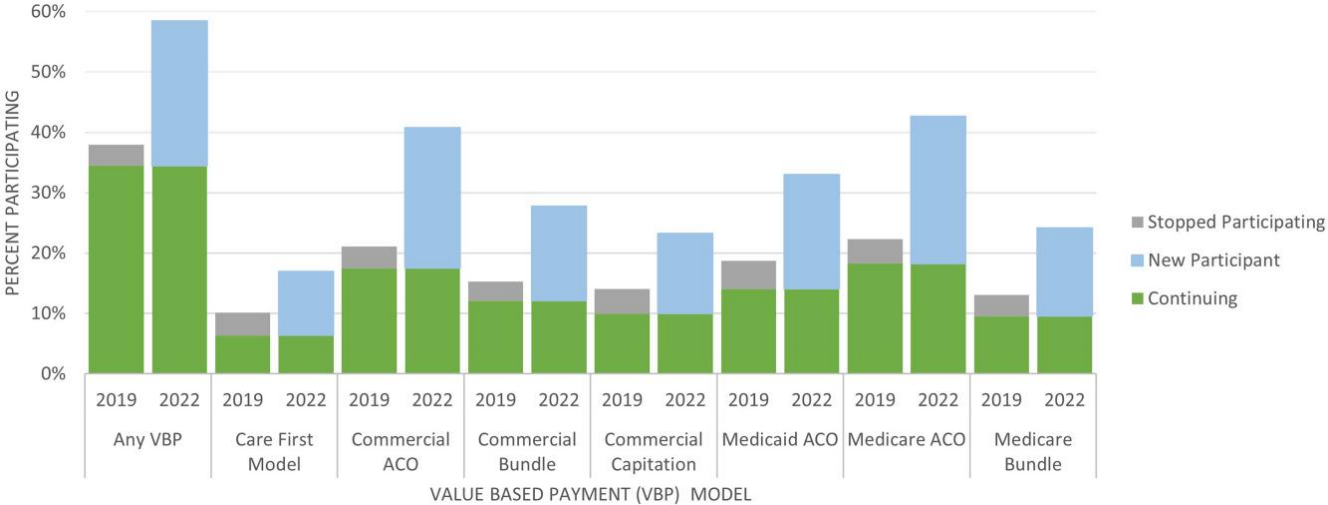
Value-based care participation in 2019 vs 2022. Source: American Association of Medical Colleges National Sample Survey of Physicians, 2022. “Stopped Participating” refers to respondents who participated in 2019 and not 2022, “New Participant” refers to respondents who participated in 2022 but not 2019, and “Continuing” were respondents who participated in 2019 and 2022. Abbreviation: ACO, Accountable Care Organization.

Among our analytical sample, 59.8% participated in VBC payment models in 2022, but only 38.6% did so in 2019 (Figure 1). The growth in participation varied across models. Medicare models and commercial ACOs exhibited the highest participation growth (24.4 and 23.3 percentage points, respectively). Among the surveyed models, care-first models had the lowest participation in 2019 and 2022 and saw the smallest growth over the 3 years (Figure 1). From 2019 and 2022, between 3.5% and 4.6% of physicians ceased their participation in specific VBC models (Figure 1).

Our cross-sectional descriptive results show that one-third (34.8%) of respondents participated in more than 1 model in 2022 (Table 1, column 2). Approximately 9% of physicians participated in every measured model. Among ACO participants, 50% participated simultaneously in all 3 ACO models (commercial, Medicare, or Medicaid) and 80% participated in ACOs from at least 2 of the 3 payer types. Similarly, 58% of bundled payment participants dually engaged in commercial and Medicare models (Appendix 2). Additionally, key physician characteristics in the cross-sectional descriptive results demonstrate differences in participation (Table 1). Notably, participation rates varied across physician specialties, with nearly 60% of PCPs, medical specialists, and hospital-based physicians participating in VBC models, while less than 40% of surgeons participate in these models (Table 1, column 3). Other independent variables with differences in participation rate include place of practice, employer, and the majority payer (Table 1, column 3). Care delivery factors differed between VBC participants and non-participants as well. For example, physicians who did not participate in telehealth participated in VBC at lower rates than telehealth users. More so, physicians who used telehealth with patients and other physicians participated in VBC models at the highest rates (Table 1, column 3). Physicians who worked with licensed mental health providers participated in VBC at higher rates than non-participants. (See Appendix 2, Table S2, for descriptive statistics of all independent variables.)

**Table 1.** Comparison of participation rates across independent variables: 2019 vs 2022.

	Number of observations (weighted proportion of sample)	Proportion of providers participating in 2022	Proportion of providers participating in 2019
All physicians	5268 (100%)	59.8%	38.6%
Specialty			
Primary	1676 (30.6%)	55.2%	39.7%
Hospital-based	1057 (21.8%)	63.6%	44.3%
Medical specialty	1131 (20.6%)	59.4%	34.9%
Other	92 (1.8%)	40.9%	38.5%
Gyn/OB	212 (4.5%)	50.4%	31.1%
Psychiatry	262 (7.0%)	49.1%	38.8%
Surgery	834 (13.7%)	36.5%	20.8%
Self-reported rurality			
Primarily urban or suburban	4388 (82.6%)	59.8%	38.9%
Mixed urban/rural	339 (5.6%)	68.8%	44.5%
Rural serving	463 (8.7%)	54.0%	32.7%
Missing	78 (3.1%)	52.5%	36.7%
Teaching status (affiliated with)			
Not teaching institution	2832 (55.0%)	58.9%	38.8%
Academic medical center or medical school	2436 (45.0%)	60.2%	37.9%
Majority payer			
Safety net	1006 (18.4%)	67.0%	40.5%
Medicare	529(8.8%)	59.5%	38.8%
Commercial	1536 (30.7%)	56.8%	39.1%
Mixed	2197 (42.0%)	58.1%	36.7%
Practice location			
Inpatient	793 (14.5%)	60.0%	39.6%
Outpatient	3442 (66.3%)	58.5%	37.3%
Emergency/urgent care	385 (7.1%)	58.1%	38.8%
Long-term	108 (1.6%)	43.8%	38.0%
Employer			
Private practice	1835 (35.6%)	55.3%	36.0%
System	598 (12.0%)	64.5%	44.0%
Hospital	969 (16.9%)	67.2%	42.2%
Group	791 (15.9%)	64.4%	41.6%
Other	439 (8.6%)	45.0%	36.5%
Multiple	636 (11.0%)	62.8%	39.8%
Census region			
South	1392 (29.2%)	56.6%	40.0%
Midwest	1127 (24.0%)	61.7%	41.2%
West	850 (17.9%)	60.9%	38.6%
Northeast	1318 (28.9%)	61.8%	38.4%
Telehealth user			
Patient and provider telehealth	1646 (31.2%)	68.3%	45.5%
Patient telehealth only	3224 (63.0%)	55.0%	35.0%
Provider telehealth only	35 (0.1%)	66.6%	43.2%
No telehealth use	363 (5.4%)	54.6%	31.2%
Work with licensed mental health providers			
Yes	1696 (32.2%)	64.7%	41.5%
No	3567 (68.8%)	58.7%	38.0%
Number of VBC models (grouped)			
0	NA	41.5%	61.4%
1	NA	23.7%	17.2%
2	NA	16.3%	11.9%
3	NA	9.7%	5.9%
4	NA	8.8%	3.7%

Abbreviations: ACO, Accountable Care Organization; Gyn/OB, gynecology/obstetrics; NA, not applicable; PCF, Primary Care First; VBC, value-based care.

Source: American Association of Medical Colleges National Sample Survey of Physicians 2022. Respondents were excluded if they worked part-time or did not answer questions on payment models. Respondents self-reported time spent in rural areas as a percentage, which we then categorized into “primarily urban or suburban” (0%-20% of time in rural areas), “mixed urban/rural time” (21%-80% of time in rural areas), or “rural serving” (81%-100% of time in rural areas). In column 2 the number of participants is the raw number and the percentage is the weighted distribution. The number of VBC models were grouped by model type: ACOs, bundled payments, capitation, and PCF models. The total number of observations for the number of models was not calculated as it depended on year of participation. Participation weights are weighted based on the same methodology as the regressions. Documentation for Sampling and Weights 2023. Available from: https://www.aamc.org/media/71861/download?attachment.

The results displayed in Table 2 tested the relationships between participation and all independent variables simultaneously to assess whether any are predictive of participation. Hospital-based physicians (odds ratio [OR] = 0.6, *P* < .001), medical specialists (OR = 0.5, *P* < .001), psychiatrists (OR = 0.4, *P* < .001), and surgeons (OR = 0.5, *P* < .001) were less likely than PCPs to participate in VBC payment models (Table 2, column 2). We did not find any statistically significant differences in participation between gynecologists/obstetricians and PCPs (Table 2, column 2).

**Table 2.** Association of physician specialty on VBC participation.

Measure, dependent variable	Odds ratio (Condfidence Interval)		Regression coefficient, number of VBC models (0–4) (Confidence Interval)
	VBC model participation	New participation in VBC model	
*n*	4685	3071	4685
Primary	Ref	Ref	Ref
Hospital-based	0.6*** (0.4, 0.8)	0.5*** (0.3, 0.8)	−0.1 (−0.3, 0.1)
Medical specialty	0.5*** (0.4, 0.7)	0.5*** (0.3, 0.7)	−0.2** (−0.4, 0.0)
Other	0.4* (0.2, 1.1)	0.3* (0.1, 1.0)	−0.4 (−0.9, 0.2)
Gyn/OB	0.8 (0.5, 1.2)	0.5* (0.3, 1.0)	0.2 (−0.2, 0.4)
Psychiatry	0.4*** (0.3, 0.7)	0.3*** (0.1, 0.6)	−0.3** (−0.6, −0.0)
Surgery	0.5*** (0.4, 0.7)	0.4*** (0.2, 0.6)	−0.3*** (−0.5, −0.1)
Control variables	Included	Included	Included

Abbreviations: ACO, Accountable Care Organization; Gyn/OB, gynecology/obstetrics; PCF, Primary Care First; ref, reference; VBC, value-based care.

Source: American Association of Medical Colleges National Sample Survey of Physicians 2022. *P < .1, **P < .05, ***P < .01. Respondents were excluded if they worked part-time or did not answer question on payment models. Control variables include: rurality, payer-mix, teaching status affiliation, place of practice, employer, employment status, race and ethnicity, gender, age, and census region. The number of VBC models were grouped by model type: ACOs, bundled payments, capitation, and PCF models. Documentation for Sampling and Weights 2023. Available from: https://www.aamc.org/media/71861/download?attachment.

We found similar results when assessing new participation since 2019. Hospital-based physicians, medical physicians, psychiatrists, and surgeons were less likely than PCPs to have recently joined any model. In 2022, medical specialists, psychiatrists, and surgeons participated in significantly fewer payment models than PCPs (Table 2, column 4). Obstetricians/gynecologists, hospital-based physicians, and other specialists did not participate in a significantly different number of models than PCPs (Table 2, column 3).

Differences between specialties emerged when analyzing individual VBC payment models. Breaking down by model type (Table 3, column 6), medical specialists (OR = 0.6, *P* < .001) and surgeons (OR = 0.6, *P* < .05) were less likely to participate in commercial capitation than PCPs. Medical specialists and obstetricians/gynecologists were more likely to participate in Medicare bundles than PCPs (Table 3, column 7). Additionally, obstetricians/gynecologists were more likely to participate in commercial bundles compared with PCPs (OR = 3, *P* < .001) (Table 3, column 8). Several other physician characteristics were associated with VBC participation. Compared with White physicians, Asian physicians were more likely to participate in VBC payment models (OR = 1.4, *P* < .05) (Appendix 2, Table S3). In particular, Asian physicians participated more in commercial capitation models, Medicare bundles, and commercial bundles (Appendix 2, Table S4). There were no statistically significant differences in participation between White physicians in comparison to Latino/Hispanic, African American/Black, mixed race, or unknown race, possibly due to small sample sizes for some categories (Appendix 2, Table S4). Mixed urban/rural physicians were more likely to participate in and recently join VBC payment models than physicians who primarily work in urban or suburban areas (*P* < .001), although they participated in 0.4 fewer payment models (*P* < .001) (Appendix 2, Table S3).

**Table 3.** Association of physician specialty on specific VBC model participation in 2022.

VBC model type	Medicare ACO	Medicaid ACO	Commercial ACO	PCF model	Commercial capitation	Medicare bundle	Commercial bundle
*n*	4685	4685	4685	4685	4685	4685	4685
Dependent variable				Odds of participation (Confidence Interval)			
Primary	Ref	Ref	Ref	Ref	Ref	Ref	Ref
Hospital-based	1.0 (0.7, 1.4)	1.0 (0.7, 1.5)	1.0 (0.7, 1.4)	0.7 (0.4, 1.1)	1.0 (0.6, 1.4)	1.6** (1.1, 2.4)	1.2 (0.8, 1.7)
Medical specialty	0.9 (0.6, 1.1)	0.9 (0.7, 1.2)	0.8 (0.6, 1.1)	0.8 (0.6, 1.2)	0.6*** (0.4,0.9)	1.1 (0.7, 1.5)	1.1 (0.8, 1.5)
Other	0.6 (0.2, 1.5)	0.6 (0.2, 1.7)	0.6 (0.2, 1.3)	0.5 (0.2, 1.5)	0.6 (0.2, 1.8)	0.7 (0.2, 2.0)	0.8 (0.3, 2.2)
Gyn/OB	0.9 (0.5. 1.5)	1.4 (0.8, 2.3)	0.8 (0.5, 1.4)	0.9 (0.5, 1.8)	1.3 (0.8, 2.4)	2.6*** (1.5, 4.6)	3*** (1.8, 5.1)
Psychiatry	0.8 (0.5, 1.4)	0.9 (0.5, 1.5)	0.7 (0.4, 1.2)	0.6 (0.3, 1.3)	0.7 (0.4, 1.3)	1.1 (0.6, 2)	0.7 (0.4, 1.3)
Surgery	0.9 (0.6, 1.2)	0.9 (0.6, 1.3)	0.9 (0.6,1.3)	0.7 (0.4, 1.1)	0.6** (0.4, 0.9)	0.8 (0.5, 1.2)	0.9 (0.6, 1.3)
Control variables	Included	Included	Included	Included	Included	Included	Included

Abbreviations: ACO, Accountable Care Organization; Gyn/OB, gynecology/obstetrics; PCF, Primary Care First; ref, reference; VBC, value-based care.

Source: American Association of Medical Colleges National Sample Survey of Physicians 2022. **P < .05, ***P < .01. Respondents were excluded if they worked part-time or did not answer question on payment models. Control variables include: rurality, payer-mix, teaching status affiliation, place of practice, employer, employment status, race and ethnicity, gender, age, and census region. Documentation for Sampling and Weights 2023. Available from: https://www.aamc.org/media/71861/download?attachment.

Compared with private practice physicians, physicians who worked in health systems were more likely to participate in VBC payment models (OR = 1.6, *P* < .001) and more likely to newly participate in VBC models (OR = 1.8, *P* < .05) (Appendix 2, Table S3). In particular, Medicare and Medicaid ACOs drove the differences in participation (Appendix 2, Table S4). Additionally, physicians working for hospitals were more likely to participate in Medicaid ACOs, physicians working for medical groups were more likely to participate in Medicare ACOs than private practice physicians (Appendix 2, Table S4). Physicians working in an emergency room or urgent care setting were more likely to participate in PCF, commercial capitation, or commercial bundle models (Appendix 2, Table S4). While academic affiliation was not significantly related to participation rates, academic-affiliated participants tended to do so across a higher number of models that non-academic physicians (0.2 models, *P* < .05) (Appendix 2, Table S3). Compared with the southern US region, physicians in the Midwest and West were more likely to newly participate in VBC models (OR = 1.5, OR = 1.7, *P* < .001) (Appendix 2, Table S3).

## Discussion

Our results highlight the substantial role that VBC plays in the current health care payment landscape, with most physicians engaging in at least 1 VBC payment model. Participation has increased across all 7 models explored in this study, showing a decreased reliance on FFS as providers embrace VBC. The high national participation rates reinforce other surveys that found similar increases in participation over time along with similar rates of participation in capitation, all types of ACOs, and any VBC models overall,^13^ although our study reported lower rates of participation in bundled payment models.^13^

These findings suggest that the types of payment models and types of employing organizations are determining factors in VBC participation. The factors influencing the intensity of participation align with those affecting the extent of participation: those most likely to participate tend to do so across multiple models. That is, participation in 1 payer's model increases the likelihood of participating in another payer's model. This finding matches previous research suggesting that certain infrastructure costs (eg, fixed data-collection/reporting expenses, nurse navigators, and data analytics) can be used across models.^22^ Therefore, by establishing systems to support VBC, physicians may find it easier to scale to more models.

Current limitations in policy and applicable models may contribute to barriers preventing VBC from being universally adopted. In particular, not all specialty types have equal opportunities to participate in VBC models. Some models center PCPs as a coordinator of care and would not be possible without primary care.^23^ While PCPs were more likely to participate in models redesigning primary care practice, they were less likely to participate in other models not designed to improve their practice or models not offered for PCPs. Obstetricians/gynecologists showed a higher likelihood of participating in capitation, which promotes coordinated care. This is consistent with payment schemes for obstetricians/gynecologists.^24^ Psychiatry tended to participate at lower rates because there are fewer VBC models available for mental health care. These findings support our hypothesis that physician specialty mediates participation through VBC models’ targeted designs and through practices with PCPs having the necessary infrastructure to participate in VBC payment models. These claims are supported by a similar study by the AMA that found that physician practices with at least some PCPs were more likely to participate in commercial, Medicare, and Medicaid ACOs compared with practices with only specialists.^13^ Our findings imply that participation in innovative payment models varies because of unequal opportunity to join. Some specialties, such as primary care, have more payment models appropriate to promote VBC. The difference in the number of payment model options available to PCPs compared with specialists may be explained by CMS and other stakeholders’ assumption that PCPs promote VBC by acting as a gatekeeper, efficiently referring to specialists, and decreasing unnecessary utilization, which guides their policy decisions. For example, the new Making Primary Care model controls referral behavior by guiding PCPs to refer to specialists based on individual utilization rates. Similarly, CMMI is designing more primary care–oriented ACO models.

Future policy should focus on increasing opportunities for specialists to participate in VBC. Specifically, making additional models for specialists would support their participation in VBC and closing the gap. These models can be developed by integrating specialists into existing or new models to increase opportunities for specialists to join in payment models to promote VBC and improve the quality of care.

In addition to individual physician factors, organizational factors such as care setting drive participation. Hospital-based physicians are less likely than others to participate in VBCs. Even within the same specialty, the place of practice was associated with different participation rates. These differences may reflect a model's targeted design, such as for episodic, acute care. Additionally, low participation rates for rural physicians reinforce the importance of community infrastructure and tools in closing inequalities in VBC adoption. Similarly, regional differences in VBC payment model participation may be explained by differences in infrastructure. Our study found that regions such as the western United States had higher rates of participation, reflecting their history of early adoption of capitation and other models, especially in California.^25^ Previous research supports the importance of resources in driving participation; specifically, higher ACO participation rates are higher among organizations in urban areas, larger entities, and those equipped with advanced health information technology.^12,18^ The concordance of VBC participation and telehealth reinforces the importance of technology infrastructure that may aid in VBC participation. Similarly, evaluations on the MSSP found that low-resourced clinics such as rural health clinics or clinics in areas with high deprivation are less likely to participate in the program.^26^ Including an equity component to VBC payment models through new outcome measures or other avenues may be a way to combat unequal VBC participation opportunity in low-resourced areas. Last, employer-level factors, such as a health system or private work setting, correlate with participation. Specifically, working in hospitals, working for a health system, or working as part of a medical group were related to higher participation. This finding is consistent with the claim that current VBC designs lack incentives for private practices. These models may be easier to adopt after consolidation, which may also drive participation and should be further examined.

## Conclusion

Despite increases in participation between 2019 and 2022, gaps in opportunities for VBC have resulted in uneven participation rates across physicians. Policymakers have numerous opportunities to better engage specialists in a true team-based care approach. Successful implementation of bundled payments and other VBC models requires building infrastructure, and individual physicians cannot alone make a successful model without the appropriate opportunities, support, and incentives. New models should also include features that appeal to providers and patients across specialties—for example, by improving outcome assessments via targeted measures developed by those specialties in registries or other avenues. Future policy should prioritize developing VBC models across the continuum of care, not only narrow fragments of it, focusing on the interface between primary and specialty care while promoting equity through increased opportunities for participation and improved design.

## Supplementary Material

qxae087_Supplementary_Data
