# Supplementary material for: Who participates in value-based care models? Physician characteristics and implications for value-based care
Source: Health Aff Sch. 2024 Jul 16;2(8):qxae087. doi: 10.1093/haschl/qxae087 (PMC11296673; doi:10.1093/haschl/qxae087)
Supplement: qxae087_Supplementary_Data [file qxae087_supplementary_data.zip › Supplemental Material.docx]

**Appendix 1: National Sample Survey of Physicians Questions**

The following are questions from the Association of American Medical Colleges’ National Sample Survey of Physicians that were used in the analysis.

Q2. Did you graduate from a medical school located in the United States (including Puerto Rico) or Canada?

- Yes
- No [SKIP NEXT QUESTION]

Q10. How do you self-identify? (Please check all that apply)

- Hispanic, Latino, or of Spanish origin
- American Indian or Alaskan Native
- Asian
- Black or African American
- Native Hawaiian or Other Pacific Islander
- White
- Other: [write-in] [ANCHOR]

Q34. During your last typical week of work (excluding any week with leave), approximately how many **hours** were you working?

- ___ **hours** – excluding call* [RANGE 0-168]

Q37.During a typical week, approximately what percent of your working time is spent in the following activities?

| **Type of Activity** | **Percent of Time** |
| --- | --- |
| Patient care |  |
| Combined patient care and teaching or training (e.g., providing patient care while supervising students or residents in clinical settings) |  |
| Teaching or training (teaching or training students or residents or other learners in non-clinical settings) |  |
| Research |  |
| Administration (including institutional service) |  |
| Other (Please specify ______________) |  |

2X2. What is your current employment status? (Please select one.)

- Employee
- Owner
- Independent contractor
- Other, please specify __________

Q54. Which of the following best describes your current main employment arrangement? (Please select all that apply.)

- Employee – medical school
- Employee – teaching hospital
- Employee – non-teaching hospital
- Employee – physician group
- Employee – government
- Employee – health system
- 10. Private practice – single specialty group
- 11. Private practice – multi-specialty group
- Private practice - solo
- Locum tenens
- Other (please describe)

2X3. What is the proportion of your patients by type of insurance? (Your best estimate is fine.)

| **Insurance type** | **Patient proportion (%)** |
| --- | --- |
| Medicare (including Medicare Advantage) or Medicare supplemental |  |
| Medicaid (including Medicaid managed care plans and CHIP) |  |
| Dual eligible (Medicare + Medicaid) |  |
| Commercially insured |  |
| Uninsured |  |
| Other (Please specify____________) |  |

2X4. In which of the following alternative payment models do you participate? And in which did you participate 3 years ago?

| **Payment model** | **Participate currently** | **Participated 3 years ago** |
| --- | --- | --- |
| Medicare ACOs | ☐ | ☐ |
| Medicaid ACOs | ☐ | ☐ |
| Commercial ACOs | ☐ | ☐ |
| Comprehensive Primary Care or Primary Care First models | ☐ | ☐ |
| Patient-centered medical homes | ☐ | ☐ |
| Medicare bundled or episode payments | ☐ | ☐ |
| Commercial bundled or episode payments | ☐ | ☐ |
| Commercial capitated arrangements | ☐ | ☐ |
| Other alternative payment model not listed above (please specify) | ☐ | ☐ |
| None/Not applicable | ☐ | ☐ |

52. Please indicate the specialty in which you currently spend the most time.

Q57. What is the zip code for the place where you spend more time providing patient care* than you do in the others?

2X5. What percentage of your patient care* time do you spend in each of the following types of places?

| **Urban** |  |
| --- | --- |
| **Suburban** |  |
| **Rural** |  |

Q55. During a typical week, approximately what percent of your patient care* time is spent in the following types of places (including visits, conducting, and reviewing tests and imaging, and performing procedures directly on patients)?

| **Type of place** | **Percent of patient care* time** |
| --- | --- |
| Ambulatory or outpatient |  |
| Urgent care |  |
| Inpatient |  |
| Emergency Department |  |
| Long Term Care |  |

Q27. Are you currently affiliated (e.g., admitting or consulting privileges) with an Academic Health Center (also called an Academic Medical Center) or teaching hospital (including paid or volunteer, full-time or part-time)?

- Yes
- No

**Appendix 2 Table 1: Model and Payer**

| Bundled Payments | | | | |
| --- | --- | --- | --- | --- |
|  | Number of Respondents | | | Percent of Those Who  Participate |
| Commercial Only | 456 | | | 27% |
| Medicare Only | 255 | | | 15% |
| Commercial and Medicare | 995 | | | 58% |
| Any Bundle | 1706 | | | 100% |
| ACOs | | | | |
|  | | Number of Respondents | Percent of Those Who  Participate | |
| Medicare Only | | 242 | 9% | |
| Medicaid Only | | 106 | 4% | |
| Commercial Only | | 176 | 7% | |
| Medicare and Medicaid | | 158 | 6% | |
| Medicare and Commercial | | 458 | 17% | |
| Commercial and Medicaid | | 178 | 7% | |
| All Three | | 1326 | 50% | |
| Any ACO | | 2644 | 100% | |

Source/Notes: SOURCE [American Association of Medical Colleges National Sample Survey of Physicians 2022: NOTES [. Respondents were excluded if they worked part time or did not answer question on payment models. Participation weights are weighted based on the same methodology as the regressions. Documentation for Sampling and Weights 2023.Available from: https://www.aamc.org/media/71861/download?attachment.]

**Appendix 2 Table 2: Characteristics Between Groups**

|  |  | **Number of Observations (Weighted Proportion of Sample)** | **Proportion of Providers Participating in 2019** | **Proportion of Providers Participating in 2022** |
| --- | --- | --- | --- | --- |
| All Physicians | | 5,268 (100%) | 38.6% | 59.8% |
| **Specialty** | Primary | 1676 (30.6%) | 39.7% | 55.2% |
|  | Hospital-Based | 1057 (21.8%) | 44.3% | 63.6% |
|  | Medical Specialty | 1131 (20.6%) | 34.9% | 59.4% |
|  | Other | 92 (1.8%) | 38.5% | 40.9% |
|  | Gyn/OB | 212 (4.5%) | 31.1% | 50.4% |
|  | Psychiatry | 262 (7.0%) | 38.8% | 49.1% |
|  | Surgery | 834 (13.7%) | 20.8% | 36.5% |
| **Age** | <40 | 967 (9.5%) | 30.4% | 55.8% |
|  | 40s | 1,426 (31.2%) | 37.4% | 62.0% |
|  | 50s | 1,415 (24.4%) | 43.1% | 64.9% |
|  | 60s | 1,185 (28.9%) | 36.9% | 54.7% |
|  | 70+ | 275 (5.9%) | 43.9% | 56.5% |
| **Gender** | Man | 62.6% | 38% | 57.7% |
|  | Woman | 37.1% | 39.7% | 63.7% |
|  | Missing | 0.0% | 19.5% | 30.4% |
| **Race or Ethnicity** | Hispanic, Latino, or of Spanish Origin | 168 (2.5%) | 39.4% | 52.8% |
|  | Asian | 1,273 (25.0%) | 40.5% | 67.6% |
|  | African  American | 127 (2.1%) | 36.4% | 55.4% |
|  | White | 3,319 (65.1%) | 38.1% | 58.8% |
|  | Other | 112 (2.4%) | 35.9% | 56.5% |
|  | Mixed | 112 (2.3%) | 41.3% | 48.7% |
|  | Unknown | 157 (0.6%) | 27.0% | 58.9% |
| **Self-Reported Rurality** | Primarily Urban or Suburban | 4,388 (82.6%) | 38.9% | 59.8% |
|  | Mixed Urban/Rural | 339 (5.6%) | 44.5% | 68.8% |
|  | Primarily Rural | 463 (8.7%) | 32.7% | 54.0% |
|  | Missing | 78 (3.1%) | 36.7% | 52.5% |
| **Majority Payer** | Safety-Net | 1,006 (18.4%) | 40.5% | 67.0% |
|  | Medicare | 529(8.8%) | 38.8% | 59.5% |
|  | Commercial | 1,536 (30.7%) | 39.1% | 56.8% |
|  | Mixed | 2,197 (42.0%) | 36.7% | 58.1% |
| **Teaching Status (affiliated with)** | Not Teaching Institution | 2,832 (55.0%) | 38.8% | 58.9% |
|  | Med School or Academic Health Center | 2,436 (45.0%) | 37.9% | 60.2% |
| **International Medical Graduate** | No | 4,471 (81.1%) | 39.2% | 59.5% |
|  | Yes | 797 (18.9%) | 27.1% | 50.9% |
| **Hours Worked** | 30-40 | 789 (16.1%) | 32.4% | 51.8% |
|  | 40-60 | 3,107 (59.7%) | 38.2% | 60.1% |
|  | 60-80 | 986 (18.7%) | 43.4% | 64.4% |
|  | 80+ | 313 (5.5%) | 37.0% | 57.4% |
| **High Patient Care User** | Not Mostly Patient Care | 801 (13.7%) | 37.6% | 55.3% |
|  | Mostly Patient Care | 4,467 (86.4%) | 38.8% | 60.2% |
| **Practice Location** | Inpatient | 793 (14.5%) | 39.6% | 60.0% |
|  | Outpatient | 3,442 (66.3%) | 37.3% | 58.5% |
|  | Emergency/Urgent Care | 385 (7.1%) | 38.8% | 58.1% |
|  | Long Term | 108 (1.6%) | 38.0% | 43.8% |
|  | Mixed | 540 (10.5%) | 38.7% | 59.6% |
| **Employment Arrangement** | Employee | 3,470 (64.2%) | 41.5% | 63.1% |
|  | Owner | 1,373 (27.2%) | 31.9% | 56.7% |
|  | Independent Contactor | 37 (7.9%) | 44.6% | 50.7% |
|  | Other | 43 (0.7%) | 37.7% | 43.8% |
| **Employer** | Private Practice | 1835 (35.6%) | 36.0% | 55.3% |
|  | System | 598 (12.0%) | 44.0% | 64.5% |
|  | Hospital | 969 (16.9%) | 42.2% | 67.2% |
|  | Group | 791 (15.9%) | 41.6% | 64.4% |
|  | Other | 439 (8.6%) | 36.5% | 45.0% |
|  | Multiple | 636 (11.0%) | 39.8% | 62.8% |
| **Telehealth User** | Patient and Provider Telehealth | 1,646 (31.2%) | 45.5% | 68.3% |
|  | Patient Telehealth Only | 3,224 (63.0%) | 35.0% | 55.0% |
|  | Provider Telehealth Only | 35 (0.1%) | 43.2% | 66.6% |
|  | No Telehealth Use | 363 (5.4%) | 31.2% | 54.6% |
| **Work With Licensed Mental Health Providers** | Yes | 1,696 (32.2%) | 41.5% | 64.7% |
|  | No | 3,567 (68.8%) | 38.0% | 58.7% |
| **Census Region** | South | 1392 (29.2%) | 40.0% | 56.6% |
|  | Midwest | 1127 (24.0%) | 41.2% | 61.7% |
|  | West | 850 (17.9%) | 38.6% | 60.9% |
|  | Northeast | 1318 (28.9%) | 38.4% | 61.8% |
| **Number of VBC Models** | 0 |  | 61.4% | 41.5% |
|  | 1 |  | 9.5% | 10.0% |
|  | 2 |  | 8.2% | 11.2% |
|  | 3 |  | 8.6% | 13.5% |
|  | 4 |  | 5.2% | 7.0% |
|  | 5 |  | 3.1% | 6.3% |
|  | 6 |  | 1.6% | 4.0% |
|  | 7 |  | 2.5% | 6.5% |
| **Number of VBC Models (Grouped)** | 0 |  | 61.4% | 41.5% |
|  | 1 |  | 17.2% | 23.7% |
|  | 2 |  | 11.9% | 16.3% |
|  | 3 |  | 5.9% | 9.7% |
|  | 4 |  | 3.7% | 8.8% |

Source/Notes: SOURCE [American Association of Medical Colleges National Sample Survey of Physicians 2022: NOTES: Respondents were excluded if they worked part time or did not answer question on payment models. Respondents self-reported time spent in rural areas as a percentage, which we then categorized into “primarily urban or suburban” (0-20% of time in rural areas), “mixed urban/rural time” (21%-80% of time in rural areas), or “primarily rural” (81%-100% of time in rural areas). In column 2 the number of participants is the raw number and the percentage is the weighted distribution. When grouping models, we grouped by model type: ACOs, bundled payments, capitation, and care first models. The total number of observations for the number of models was not calculated as it depended on year of participation. Participation weights are weighted based on the same methodology as the regressions. Documentation for Sampling and Weights 2023. Available from: https://www.aamc.org/media/71861/download?attachment.]

**Appendix 2 Table 3: Association of Physician Characteristics on VBC Model Participation**

|  |  | **VBC Model Participation (n=4,685)** | **New Participation in VBC Model (n=3,071)** | **Number of VBC Models (n=4,685)** |
| --- | --- | --- | --- | --- |
| **Specialty** | Primary | ref | ref | ref |
|  | Hospital-Based | 0.6*** (0.4, 0.8) | 0.5*** (0.3, 0.8) | -0.1 (-0.3, 0.1) |
|  | Medical Specialty | 0.5*** (0.4, 0.7) | 0.5*** (0.3, 0.7) | -0.2** (-0.4, 0.0) |
|  | Other | 0.4* (0.2, 1.1) | 0.3* (0.1, 1.0) | -0.4 (-0.9, 0.2) |
|  | Gyn/OB | 0.8 (0.5, 1.2) | 0.5* (0.3, 1.0) | 0.2 (-0.2, 0.4) |
|  | Psychiatry | 0.4*** (0.3, 0.7) | 0.3*** (0.1, 0.6) | -0.3** (-0.6, -0.0) |
|  | Surgery | 0.5*** (0.4, 0.7) | 0.4*** (0.2, 0.6) | -0.3*** (-0.5, -0.1) |
| **Age** | <40 | ref | ref | ref |
|  | 40s | 1.4* (1.0, 2.1) | 1.3 (0.8, 2.1) | 0.1 (-0.2, 0.3) |
|  | 50s | 1.5* (1.0, 2.2) | 1.1 (0.7, 1.9) | 0.1 (-0.1, 0.4) |
|  | 60s | 1.1 (0.8, 1.7) | 0.9 (0.6, 1.6) | -0.1 (-0.3, 0.2) |
|  | 70+ | 1.2 (0.7, 2.1) | 0.6 (0.3, 1.3) | -0.2 (-0.5, 0.2) |
| **Gender** | Men | ref | Ref | ref |
|  | Women | 1.1 (0.9, 1.4) | 1.2 (0.9, 1.7) | 0.1 (-0.1, 0.2) |
|  | Missing | 0.4 (0.1, 2.4) | 0.2** (0, 0.7) | -0.3 (-1, 0.4) |
| **Race or Ethnicity** | Hispanic, Latino, or of Spanish Origin | 1 (0.5, 2) | 1.2 (0.5, 3.0) | 0.1 (-0.4, 0.5) |
|  | Asian | 1.4** (1, 1.8) | 1.5** (1.1, 2.1) | 0.2*** (0.1, 0.4) |
|  | Black or African American | 0.9 (0.4, 1.9) | 1 (0.4, 2.5) | 0 (-0.4, 0.5) |
|  | White | ref | ref | ref |
|  | Other | 0.8 (0.4, 1.5) | 0.7 (0.3, 1.7) | 0.1 (-0.3, 0.5) |
|  | Mixed | 0.6 (0.3, 1.2) | 0.2*** (0, 0.6) | -0.2 (-0.7, 0.3) |
|  | Unknown | 0.9 (0.3, 3.4) | 0.8 (0.1, 4.7) | 0.1 (-0.5, 0.6) |
| **Rurality** | Primarily Urban or Suburban | ref | ref | ref |
|  | Mixed Urban/Rural | 2.1*** (1.2, 3.5) | 2.3** (1.1, 4.6) | 0.4*** (0.1, 0.7) |
|  | Primarily Rural | 0.7* (0.5, 1.0) | 0.8 (0.5, 1.3) | -0.2** (-0.4, 0.0) |
|  | Missing | 0.8 (0.4, 1.4) | 0.8 (0.4, 1.8) | -0.1 (-0.4, 0.2) |
| **Majority-Payor** | Mixed | ref | ref | ref |
|  | Safety-Net | 1.2 (0.9, 1.7) | 1.4* (1.0, 2.1) | 0.2** (0.0, 0.4) |
|  | Medicare | 1.2 (0.8, 1.8) | 1.5 (0.9, 2.5) | 0.1 (-0.1, 0.4) |
|  | Commercial | 1.14 (0.9, 1.5) | 1.1 (0.8, 1.6) | 0.2 (0.0, 0.3) |
| **Teaching Status** | Not Teaching Institution | ref | ref | ref |
|  | Med School or Academic Health Center | 1 (0.8, 1.3) | 1.3 (0.9, 1.7) | 0.2** (0.0, 0.3) |
| **International Medical Graduate** | US-Training | ref | ref | ref |
|  | International Medical Graduate | 1.4** (1.0, 1.9) | 1.4* (1.0, 2.1) | 0.3*** (0.1, 0.5) |
| **Hours Worked** | 30-40 | ref | ref | ref |
|  | 40-60 | 1.4** (1.0, 1.9) | 1.4 (0.9, 2.0) | 0.2* (0.0, 0.3) |
|  | 60-80 | 1.7*** (1.2, 2.5) | 1.5* (0.9, 2.5) | 0.3*** (0.1, 0.5) |
|  | 80+ | 1.2 (0.7, 2.1) | 1.9*(0.9, 3.6) | 0.3* (-0.0, 0.7) |
| **High Patient Care User** | Not Mostly Patient Care | ref | ref | ref |
|  | Mostly Patient Care | 1.3 (0.9 1.7) | 1.1 (0.7, 1.7) | 0.2 (-0.0, 0.3) |
| **Practice Location** | Inpatient | ref | ref | ref |
|  | Outpatient | 1.1 (0.7, 1.5) | 1.5* (0.9, 2.5) | 0.1 (-0.2, 0.3) |
|  | Emergency/Urgent Care | 1.2 (0.7, 2.0) | 1.3 (0.7, 2.6) | 0.4** (0.0, 0.7) |
|  | Long Term | 0.6 (0.3, 1.2) | 0.7 (0.2, 2.4) | -0.3 (-0.7, 0.2) |
|  | Mixed | 1.3 (0.8, 2) | 2.1** (1.2, 4) | 0.3** (0.0, 0.6) |
| **Employer Status** | Employee | ref | ref | ref |
|  | Owner | 0.9 (0.6, 1.3) | 1 (0.6, 1.6) | -0.1 (-0.3, 0.1) |
|  | Independent Contactor | 0.8 (0.5, 1.3) | 1 (0.6, 1.8) | -0.2 (-0.4, 0.1) |
|  | Other | 0.4 (0.1, 1.5) | 1.3 (0.4, 4.5) | -0.5** (-1, -0.1) |
| **Employment Arrangement** | Private Practice | ref | ref | ref |
|  | System | 1.6** (1.0, 2.6) | 1.8** (1.0, 3.3) | 0.1 (-0.1, 0.4) |
|  | Hospital | 1.5* (1.0, 2.4) | 1.6 (0.9, 3.0) | 0.2* (-0.0, 0.5) |
|  | Group | 1.4 (0.9, 2.1) | 1.3 (0.8, 2.2) | 0.2 (-0.1, 0.4) |
|  | Other | 0.7 (0.4, 1.2) | 0.6* (0.3, 1.1) | -0.2 (-0.5, 0.1) |
|  | Multiple | 1.3 (0.8, 2.0) | 1.1 (0.6, 2.1) | 0.1 (-0.2, 0.3) |
| **Census Region** | South | ref | ref | ref |
|  | Midwest | 1.3* (1.0, 1.7) | 1.5** (1.0, 2.2) | 0.1 (-0.1, 0.3) |
|  | West | 1.4* (1.0, 1.9) | 1.7** (1.1, 2.5) | 0.2 (-0.0, 0.3) |
|  | Northeast | 1.2 (0.9, 1.6) | 1.2 (0.9, 1.8) | 0 (-0.2, 0.2) |

SOURCE [American Association of Medical Colleges National Sample Survey of Physicians 2022.]

NOTES [*p<0.1, **p<0.05, ***p<0.01. Respondents were excluded if they worked part time or did not answer question on payment models. Respondents self-reported time spent in rural areas as a percentage, which we then categorized into “primarily urban or suburban” (0-20% of time in rural areas), “mixed urban/rural time” (21%-80% of time in rural areas), or “primarily rural” (81%-100% of time in rural areas). The number of VBC models were grouped by model type: ACOs, bundled payments, capitation, and care first models. Control variables include: rurality, payer-mix, teaching status affiliation, place of practice, employer, employment status, race, gender, age, and census region. Documentation for Sampling and Weights 2023 available from: https://www.aamc.org/media/71861/download?attachment.]

**Appendix 2 Table 4. Association of Physician Characteristics on Specific VBC Model Participation in 2022**

|  | **VBC Model Type** | **Medicare ACO** | **Medicaid ACO** | **Commercial ACO** | **PCF**  **Model** | **Commercial Capitation** | **Medicare Bundle** | **Commercial Bundle** |
| --- | --- | --- | --- | --- | --- | --- | --- | --- |
|  | n= | 4,685 | 4,685 | 4,685 | 4,685 | 4,685 | 4,685 | 4,685 |
|  | **Dependent variable** | Odds of Participation | | | | | | |
| **Specialty** | Primary | ref | ref | ref | ref | ref | ref | ref |
|  | Hospital-Based | 1.0  (0.7, 1.4) | 1.0  (0.7, 1.5) | 1.0  (0.7, 1.4) | 0.7  (0.4, 1.1) | 1.0  (0.6, 1.4) | 1.6**  (1.1, 2.4) | 1.2  (0.8, 1.7) |
|  | Medical Specialty | 0.9  (0.6, 1.1) | 0.9  (0.7, 1.2) | 0.8  (0.6, 1.1) | 0.8  (0.6, 1.2) | 0.6***  (0.4,0.9) | 1.1  (0.7, 1.5) | 1.1  (0.8, 1.5) |
|  | Other | 0.6  (0.2, 1.5) | 0.6  (0.2, 1.7) | 0.6  (0.2, 1.3) | 0.5  (0.2, 1.5) | 0.6  (0.2, 1.8) | 0.7  (0.2, 2) | 0.8  (0.3, 2.2) |
|  | Gyn/OB | 0.9  (0.5. 1.5) | 1.4  (0.8, 2.3) | 0.8  (0.5, 1.4) | 0.9  (0.5, 1.8) | 1.3  (0.8, 2.4) | 2.6***  (1.5, 4.6) | 3***  (1.8, 5.1) |
|  | Psychiatry | 0.8 (0.5, 1.4) | 0.9 (0.5, 1.5) | 0.7 (0.4, 1.2) | 0.6  (0.3, 1.3) | 0.7  (0.4, 1.3) | 1.1  (0.6, 2) | 0.7  (0.4, 1.3) |
|  | Surgery | 0.9  (0.6, 1.2) | 0.9  (0.6, 1.3) | 0.9  (0.6,1.3) | 0.7  (0.4, 1.1) | 0.6**  (0.4, 0.9) | 0.8  (0.5, 1.2) | 0.9  (0.6, 1.3) |
| **Age** | <40 | ref | ref | ref | ref | ref | Ref | Ref |
|  | 40s | 1.0  (0.7, 1.5) | 1.2  (0.8, 1.8) | 1.1  (0.7, 1.6) | 1.0  (0.6, 1.5) | 0.9  (0.6, 1.5) | 1.2  (0.8, 2) | 1.0  (0.7, 1.6) |
|  | 50s | 1.1  (0.7, 1.6) | 1.2  (0.8, 1.8) | 1.2  (0.8, 1.7) | 0.7  (0.5, 1.2) | 1.2  (0.8, 2) | 1.4  (0.9, 2.3) | 1.4  (0.9, 2.1) |
|  | 60s | 0.7  (0.5, 1.1) | 0.9  (0.6, 1.3) | 0.8  (0.5, 1.2) | 0.6**  (0.3, 0.9) | 0.9  (0.5, 1.4) | 0.9  (0.5, 1.4) | 0.8  (0.5, 1.3) |
|  | 70+ | 0.9  (0.5, 1.5) | 0.9  (0.5, 1.6) | 0.8  (0.4, 1.3) | 0.5*  (0.2, 1.1) | 1.0  (0.5, 2) | 0.7  (0.3, 1.6) | 0.8  (0.4, 1.6) |
| **Gender** | Men | ref | ref | ref | ref | ref | ref | ref |
|  | Women | 1.0  (0.8, 1.3) | 1.2  (0.9, 1.5) | 0.9  (0.7, 1.1) | 1.0  (0.7, 1.4) | 1.0  (0.8, 1.4) | 1.4**  (1, 1.8) | 1.1  (0.8, 1.4) |
|  | Missing | 0.8  (0.1, 5.1) | 1.6  (0.3, 9.6) | 0.1*** (0, 0.3) | 0.2**  (0.1, 0.9) | 0.1***  (0, 0.3) | 2.3  (0.3, 15.9) | 1.2  (0.1, 10.6) |
| **Race** | Hispanic, Latino, or of Spanish Origin | 1.1 (0.6, 2.3) | 0.9 (0.5, 1.8) | 1.0 (0.5, 2) | 1.2 (0.5, 3.0) | 1.2 (0.5, 2.6) | 1.0  (0.4, 2.4) | 0.8  (0.4, 1.8) |
|  | Asian | 1.1  (0.8, 1.4) | 1.1  (0.8, 1.5) | 1.1  (0.9, 1.5) | 1.4*  (1, 1.9) | 1.4**  (1.1, 2.0) | 1.6***  (1.2,2.2) | 1.5***  (1.1, 2.0) |
|  | Black or African American | 1.1  (0.5, 2.2) | 1.1  (0.5, 2.2) | 1.1  (0.5, 2.3) | 1.2  (0.5, 3.2) | 0.7  (0.3, 1.7) | 1.2  (0.5, 2.7) | 0.8  (0.3, 2) |
|  | White | ref | ref | ref | ref | ref | ref | ref |
|  | Other | 1.4  (0.7, 2.8) | 0.7  (0.3, 1.4) | 1.6  (0.8, 3) | 1.2  (0.5, 2.8) | 1.6  (0.7, 3.7) | 1.3  (0.5, 3) | 0.8  (0.3, 1.8) |
|  | Mixed | 0.6  (0.3, 1.4) | 0.8  (0.3, 1.8) | 0.8  (0.4, 1.7) | 0.7  (0.2, 2.3) | 0.8  (0.3, 2.2) | 0.7  (0.3, 1.9) | 1.0  (0.4, 2.4) |
|  | Unknown | 2.1  (0.6 6.5) | 2.2  (0.8, 6.4) | 0.9  (0.3, 2.6) | 0.2***  (0.1, 0.4) | 0.9  (0.3, 2.7) | 3.1**  (1.1, 9.1) | 1.8  (0.7, 5.0) |
| **Rurality** | Primarily Urban or Suburban | ref | ref | ref | ref | ref | ref | ref |
|  | Mixed Urban/Rural | 1.4  (0.8, 2.2) | 2.0**  (1.2, 3.3) | 1.7**  (1.1, 2.7) | 1.4  (0.8, 2.5) | 1.9**  (1.1, 3.2) | 2.0**  (1.1, 3.2) | 2.0***  (1.2,3.3) |
|  | Primarily Rural | 0.8  (0.6, 1.2) | 1.1  (0.8, 1.6) | 1  (0.7, 1.4) | 0.7  (0.4, 1.2) | 0.6**  (0.3, 0.9) | 0.9  (0.6, 1.4) | 0.7  (0.5, 1.2) |
|  | Missing | 0.5**  (0.3, 0.8) | 0.7  (0.4, 1.4) | 0.7  (0.4, 1.3) | 0.7  (0.3,1.8) | 0.7  (0.3, 1.3) | 1.2  (0.6, 2.2) | 0.7  (0.4, 1.5) |
| **Majority Payer** | Mixed | ref | ref | ref | ref | ref | ref | ref |
|  | Safety-Net | 1.4**  (1.1, 1.9) | 1.1  (0.8, 1.5) | 1.1  (0.8, 1.4) | 1.4  (0.9, 2.0) | 1.2  (0.9, 1.7) | 1.8***  (1.3, 2.4) | 1.2  (0.9, 1.7) |
|  | Medicare | 1.3  (0.9, 1.9) | 0.7*  (0.4, 1.0) | 0.8  (0.5, 1.2) | 0.9  (0.5, 1.6) | 1.2  (0.7, 2) | 1.3  (0.8, 2.2) | 1.0  (0.6, 1.7) |
|  | Commercial | 0.8 (0.6, 1.1) | 0.7** (0.5, 0.9) | 1.2  (0.9, 1.5) | 1.3  (0.9, 1.9) | 1.8  (1.3, 2.5) | 1.0  (0.7, 1.3) | 1.5***  (1.1, 2.1) |
| **Teaching Status** | Not Teaching Institution | ref | ref | ref | ref | ref | ref | ref |
|  | Med School or Academic Health Center | 1.0  (0.8, 1.3) | 1.2  (0.9, 1.5) | 1.3**  (1.1,1.7) | 1.3*  (1.0, 1.8) | 1.2  (0.9, 1.6) | 1.4**  (1.0, 1.8) | 1.4***  (1.1,1.8) |
| **International Medical Graduate** | US-Training | ref | ref | ref | ref | ref | ref | ref |
|  | International Medical Graduate | 1.4**  (1.0, 1.9) | 1.7***  (0.2,2.0) | 1.3*  (1.0, 1.8) | 2.1***  (1.4, 3.0) | 1.3  (0.9, 1.8) | 1.3  (0.9, 1.8) | 1.2  (0.9, 1.7) |
| **Hours Worked** | 30-40 | ref | ref | ref | ref | ref | ref | ref |
|  | 40-60 | 1.5**  (1.1, 2.0) | 1.1  (0.8, 1.5) | 1.1  (0.8, 1.4) | 1.2  (0.8, 1.9) | 1.2  (0.8, 1.7) | 1.4  (0.9, 2.1) | 1.3  (0.7, 1.6) |
|  | 60-80 | 1.7***  (1.2,2.5) | 1.1  (0.7, 1.6) | 1.2  (0.8, 1.7) | 1.5  (0.9, 2.4) | 1.4  (0.9, 2.1) | 2.0***  (1.3, 3.2) | 1.5**  (1.0, 2.3) |
|  | 80+ | 1.3  (0.8, 2.3) | 1.0  (0.5, 1.7) | 0.9  (0.5, 1.6) | 1.5  (0.8. 3) | 2.4***  (1.3, 4.4) | 2.4***  (1.3, 4.3) | 2.0**  (1.1, 3.7) |
| **Practice Location** | Inpatient | ref | ref | ref | ref | ref | Ref | Ref |
|  | Outpatient | 1.2  (0.8, 1.7) | 1.0  (0.7, 1.4) | 1.0  (0.7, 1.4) | 1.4  (0.8, 2.2) | 1.2  (0.8, 1.9) | 1.0  (0.7, 1.4) | 1.1  (0.7, 1.6) |
|  | Emergency/  Urgent Care | 1.2  (0.7, 2) | 1.2  (0.7, 2.2) | 1.0  (0.6, 1.7) | 2.3**  (1.2, 4.4) | 2.2**  (1.2, 4) | 1.7*  (1, 3.1) | 1.9**  (1,1, 3.4) |
|  | Long Term | 0.5  (0.2, 1.3) | 0.6  (0.2, 1.8) | 0.4*  (0.2, 1.1) | 0.8  (0.2, 2.7) | 0.5  (0.1, 2.1) | 0.6  (0.1, 2.3) | 1.4  (0.5, 3.7) |
|  | Mixed | 1.4  (0.9, 2.2) | 0.9  (0.6, 1.5) | 1.0  (0.6, 1.6) | 2.0**  (1.1, 3.5) | 2.1***  (1.2, 3.5) | 1.7**  (1.0, 2.8) | 1.6**  (1.0, 2.6) |
| **Employment Arrangement** | Employee | ref | ref | ref | ref | ref | ref | ref |
|  | Owner | 0.8  (0.5, 2.5) | 0.8  (0.5, 1.2) | 0.9  (0.6, 1.3) | 0.7  (0.4, 1.1) | 0.7  (0.5, 1.3) | 1.0  (0.6, 1.5) | 0.9  (0.6, 1.3) |
|  | Independent Contactor | 0.8 (0.5,1.2) | 0.9  (0.5, 1.4) | 0.8  (0.5, 1.3) | 0.6  (0.3, 1.2) | 0.8  (0.4, 1.4) | 0.9  (0.5, 1.5) | 0.8  (0.5, 1.4) |
|  | Other | 0.1  (0.0, 1.0) | 0.5  (0.1, 1.8) | 0.2**  (0.1, 0.9) | 0.0***  (0, 0.1) | 0.2  (0.0, 1.6) | 1.6  (0.4, 6.0) | 0.4  (0.1, 2) |
| **Employer** | Private Practice | ref | ref | ref | ref | ref | ref | ref |
|  | System | 1.6** (1.0, 2.5) | 1.8**  (1.1, 2.9) | 1.3  (0.9, 2.1) | 1.1  (0.6, 2) | 0.8  (0.4, 1.3) | 1.5  (0.9, 2.6) | 1.3  (0.8, 2.2) |
|  | Hospital | 1.3  (0.9, 2.1) | 1.9***  (1.2, 3.0) | 1.4  (0.9, 2.2) | 1.5  (0.8, 2.6) | 1.0  (0.6, 1.7) | 1.6*  (1.0, 2.8) | 1.4  (0.9 2.4) |
|  | Group | 1.6**  (1.0, 2.4) | 1.5*  (1.0, 2.3) | 1.5*  (1.0, 2.2) | 1.1  (0.7, 1.9) | 1.0  (0.6, 1.7) | 1.2  (0.7, 2.0) | 1.3  (0.8, 2.1) |
|  | Other | 0.7  (0.4, 1.1) | 1.0  (0.6, 1.8) | 0.6*  (0.4, 1.1) | 0.8  (0.4, 1.5) | 0.6*  (0.3, 1.1) | 1.0  (0.6, 1.9) | 0.8  (0.5, 1.5) |
|  | Multiple | 1  (0.6, 1.5) | 1.4  (0.9, 2.3) | 1.5*  (1, 2.3) | 1  (0.6, 1.8) | 0.9  (0.5, 1.6) | 1  (0.5, 1.7) | 1.3  (0.8, 2.1) |
| **High Patient Care User** | Not Mostly Patient Care | ref | ref | ref | ref | ref | ref | ref |
|  | Mostly Patient Care | 1.4**  (1, 2) | 1.2  (0.8, 1.6) | 1.5**  (1.1, 2.0) | 1.1  (0.7, 1.7) | 1.3  (0.8, 1.9) | 1.4*  (0.9, 2.1) | 1.0  (0.7, 1.5) |
| **Census Region** | South | ref | ref | ref | ref | ref | ref | ref |
|  | Midwest | 1.2  (0.9, 1.6) | 1.1  (0.8, 1.5) | 1.0  (0.7, 1.3) | 0.8  (0.5, 1.1) | 1.3  (0.9, 1.8) | 1.2  (0.9, 1.7) | 1.2  (0.9, 1.7) |
|  | West | 1.0  (0.8, 1.4) | 1.1  (0.8, 1.6) | 1.0  (0.7, 1.3) | 1.2  (0.8, 1.9) | 1.7***  (1.2, 2.5) | 1.1  (0.7, 1.6) | 1.1  (0.8, 1.5) |
|  | Northeast | 1.1  (0.9, 1.5) | 1.2  (0.9, 1.5) | 0.8  (0.6, 1.1) | 0.9  (0.6, 1.3) | 1.1  (0.8, 1.6) | 1.1  (0.8, 1.6) | 1.0  (0.7, 1.4) |

SOURCE [American Association of Medical Colleges National Sample Survey of Physicians 2022.]

NOTES [*p<0.1, **p<0.05, ***p<0.01. Respondents were excluded if they worked part time or did not answer question on payment models. Respondents self-reported time spent in rural areas as a percentage, which we then categorized into “primarily urban or suburban” (0-20% of time in rural areas), “mixed urban/rural time” (21%-80% of time in rural areas), or “primarily rural” (81%-100% of time in rural areas) Control variables include: rurality, payer-mix, teaching status affiliation, place of practice, employer, employment status, race/ethnicity, gender, age, and census region. Documentation for Sampling and Weights 2023 available from: https://www.aamc.org/media/71861/download?attachment.]
